# Sports Specialization and Sports-Related Injuries in Japanese School-Aged Children and Adolescents: A Retrospective Descriptive Study

**DOI:** 10.3390/ijerph18147369

**Published:** 2021-07-09

**Authors:** Ryosuke Shigematsu, Shuta Katoh, Koya Suzuki, Yoshio Nakata, Hiroyuki Sasai

**Affiliations:** 1 Faculty of Education, Mie University, Tsu 514-8507, Japan; 2 Course for Health and Physical Education, Faculty of Education, Mie University, Tsu 514-8507, Japan; ka.shu1.tch@gmail.com; 3 Department of Sports Science, Juntendo University, Chiba 270-1695, Japan; ko-suzuki@juntendo.ac.jp; 4 Faculty of Health and Sport Sciences, University of Tsukuba, Tsukuba 305-8577, Japan; nakata.yoshio.gn@u.tsukuba.ac.jp; 5 Research Team for Promoting Independence and Mental Health, Tokyo Metropolitan Institute of Gerontology, Tokyo 173-0015, Japan; sasai@tmig.or.jp

**Keywords:** extra-curriculum, early specialization, athletic injuries

## Abstract

Although early sports specialization is associated with sports-related injuries, relevant quantitative studies on young non-elite athletes, the majority of sports participants, are scarce. We described sports specialization time points and the characteristics of sports-related injuries. Undergraduate students at a university in Japan (*n* = 830) recalled their history of sports participation from elementary to high school and sports-related injuries in a self-administered questionnaire. Of 570 valid respondents, 486 (85%) engaged in sports at least once. Significantly more respondents played multiple sports in upper elementary school (30%) than in other school categories (1–23%). In junior high and high schools, 90% and 99% played only one sport, respectively. Of the 486 respondents who played sports, 263 (54%) had experienced acute or overuse injuries. The proportion of injured participants significantly differed by school category: lower elementary school (4%), upper elementary school (21%), junior high (35%), and high school (41%). The proportions of acute or overuse injuries in males were higher than those in females. In conclusion, this study clarified a slight variation in sports items, particularly in junior high and high schools, which demonstrates 13 years as the age of beginning specialization in a single sport. More than half of the non-elite athletes experienced sports-related injuries. Injuries were frequently observed in males and those in junior high and high schools.

## 1. Introduction

As of 2015, in Japan, 57.3% of students aged 10–19 years participated in extra-curricular activities in schools (called Bukatsu in Japanese) or community/commercial-based sports clubs [1]. Sports activities are accompanied by sports-related injuries, which are affected by various factors, such as age, skeletal maturity, strength, range of motion, and skill level [2].

The amount of time spent on sports is a risk factor for sports-related injuries [3]. A case–control study revealed that young athletes aged between 7 and 18 years who were trained in weekly hours exceeding their age were significantly more likely to be injured [4]. Sports specialization is another potential risk factor [5], which occurs when athletes participate in a single sport and often year-round [6]. Sports specialization leads to overuse injuries of the shoulder, elbow, or other joints [2,7]. However, young athletes believe that specialization increases their chances of getting injured (45.8% of the entire sample), with 90.0% and 80.9% believing that specialization increases their chances of performing better in sports and making their high school team, respectively [8]. Previous studies have reported that early specialization could frequently lead to sports-related injuries, menstrual disorders, and severe psychological stress [6,9,10]. In contrast, late specialization, such as between 15 and 16 years of age, could reduce these adverse events [6]. Re-injury could frequently occur [4,11]; therefore, early sports specialization should be avoided.

In Japan, although previous studies have reported the prevalence of sports-related injuries in elite athletes on certain sports items [12,13], quantitative data on sports-related injuries in non-elite athletes, who constitute the majority of athletes, remain underreported. Risk factors for sports-related injuries may be different between non-elite and elite athletes; thus, robust research could benefit non-elite athletes and provide actionable insights for sports-related policymakers. Therefore, the present study makes an effort to ascertain when school-aged children and adolescents should begin specializing in a single sport and investigate the detailed characteristics of sports-related injuries.

## 2. Materials and Methods

### 2.1. Study Design and Participants

This retrospective descriptive study used an anonymous questionnaire. We collected data from a convenience sample from May to July 2017 and analyzed the data from August 2017 to March 2020.

**Figure 1:**
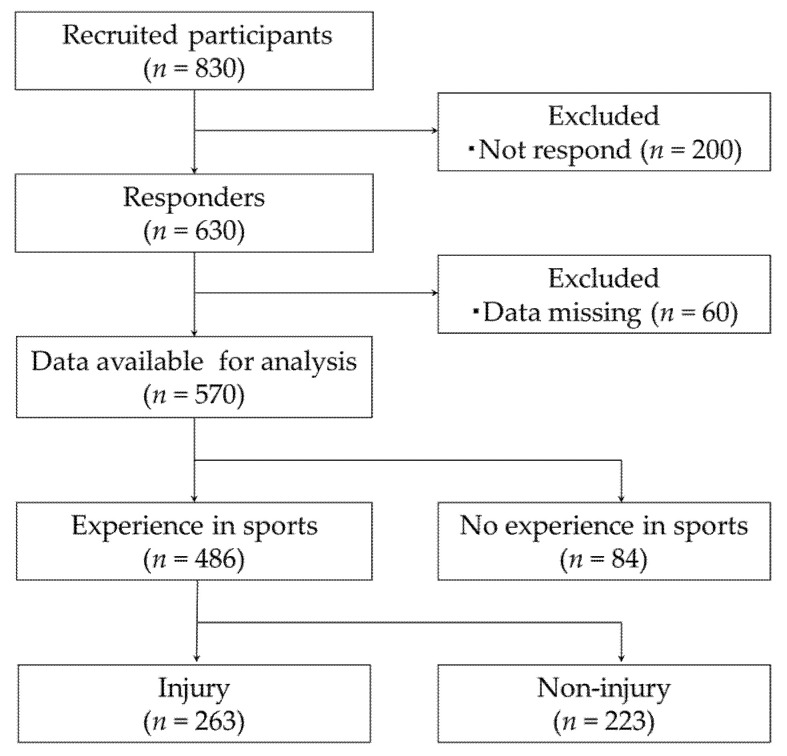
Flowchart of the selection of study participants.

Recruited participants (*n* = 830) were undergraduate freshmen to senior year students in the Faculty of Education, Mie University, Japan. Mie University is a national university located in the central part of Japan. None of the participants passed the university entrance examination based solely on sports performance. We explained the nature and purpose of the study to all participants who enrolled and asked them to participate. Following the acquisition of their written consent, we requested them to complete a questionnaire that could precisely record past sports activities and sports-related injuries. The questionnaire was developed based on previous studies [14,15,16]. The filled questionnaire was placed in an envelope to conceal their identities. Of the 830 potential participants, 630 responded to the questionnaire (76% response proportion), of whom 60 were excluded due to missing data on injury in the chronological tables. Finally, 570 participants were analyzed (69% response proportion) (Figure 1).

The study was conducted in accordance with the Declaration of Helsinki, and the ethical research committee of Mie University Faculty of Education approved the study protocol (No. 2017-7). We retrospectively registered the study protocol in the UMIN Clinical Trials Registry (UMIN 000036748).

### 2.2. Characteristics

The participants disclosed their demographic and somatometric parameters, such as sex, age, height, and weight, in the questionnaire. Body mass index (BMI) was calculated as weight in kilograms divided by height in meters squared. We also asked them to report their sports-starting age, which was defined as the age when participants started playing any sports in their lives.

### 2.3. Sports Experiences

Sports experiences were defined as activities conducted at any sports organization, such as sports clubs and extra-curricular activities (Bukatsu); however, we excluded physical education classes in schools and other recreational activities [16].

The questionnaire included a chronological table to minimize recall bias and enable participants to write their sports experiences in detail (Table S1). In the table, the participants responded to questions regarding sports items, years/months, session frequency per week, hours per session, and best performance level (national, prefecture, and city-level competitions) [14,15,16]. In addition, we listed sports items according to the Japan Olympic Committee [17] or sports organizations. Weekly hours spent in sports were defined as the product of session frequency per week and hours per session.

### 2.4. Sports-Related Injuries

We described a sports-related injury as an acute or overuse injury that occurred during—or was caused by—sports, where a given student could not participate in training or competition for one day or longer. In the definition, we included both undiagnosed and diagnosed injuries by physicians. Acute injuries were defined as those caused by a single traumatic event, such as a collision, twist, or overstretching [18]. Injuries that could not be attributed to such an event but resulted from a high repetitive motion sequence and a single discipline load pattern were classified as overuse injuries [18]. Similar to sports experiences, the participants filled in information regarding all injuries into chronological tables, including information on acute/overuse, injured part of the body, recovery duration (including self-estimation), returning to sports before and after recovery, and re-injury [14,16]. If an injury had not been healed by the end of the high school period, the duration until graduation from high school was adopted.

### 2.5. Statistical Analysis

The data were divided according to school category: lower elementary school (grades 1–3 or ages 7–9 years), upper elementary school (grades 4–6 or ages 10–12 years), junior high school (grades 1–3 or ages 13–15 years), and high school (grades 1–3 or ages 16–18 years).

The exact binomial, Fisher’s exact, or chi-square tests were used to compare the proportions. The exact binomial test was used as a post hoc test when the chi-square test was significant. We used the Kolmogorov–Smirnov test to examine the normal distribution of the data. If the data were non-normally distributed, the median and interquartile range (IQR) were calculated. When the data were normally distributed, the mean and standard deviation (SD) were calculated. Unpaired *t*-test or Wilcoxon rank-sum test was used to find significant differences in questionnaire data such as height, weight, and sports duration. One-way analysis of variance was used to detect significant differences among the school categories. Scheffe’s post hoc test was adopted.

All analyses were performed using IBM SPSS Statistics 25 or R (ver. 3.6.3) [19]. A *p*-value of <0.05 was considered statistically significant.

## 3. Results

### 3.1. Participants and Their Characteristics

The participants aged from 18 to 24 years. The ratio of male to female respondents was 42%/58%, which was not significantly different from the potential participants (40%/60%). The participants’ characteristics are summarized in Table 1, demonstrating that male participants were significantly larger in height, weight, and BMI than the national averages, whereas female participants were smaller in weight and BMI than the national averages [20].

### 3.2. Sports Experience and Specialization

**Figure 2:**
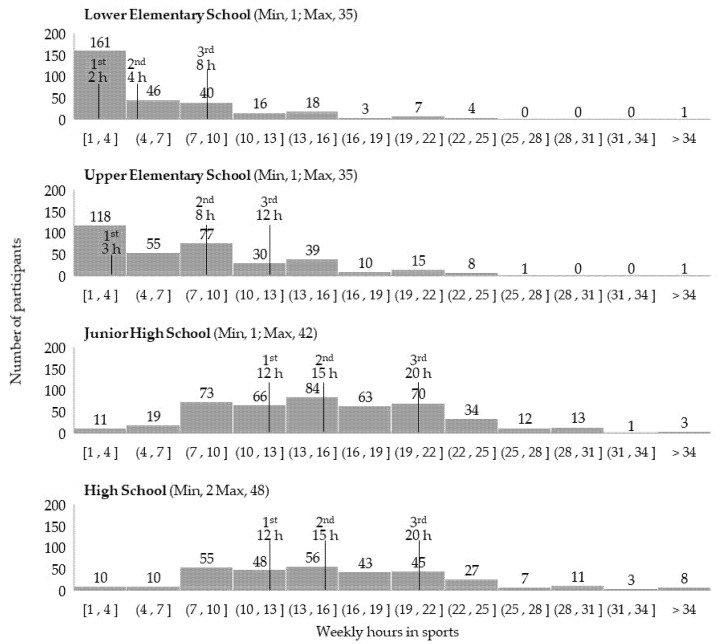
Distributions of weekly hours in sports by school category. 1st, 2nd, and 3rd: First to third quartile of weekly hours, (x, y]: x or longer, but less than y.

A total of 486 (85% of the entire analytic sample) experienced any sports in their lifetime (across lower elementary to high school) (Table 2, Table S2). Among them, 142 (29%) participated in one sports item, and 344 (71%) participated in two or more items. Of the 344 participants, 166 (34%) participated in two, 108 (22%) participated in three, and 70 (14%) participated in four or more items. More than half of the participants experienced sports in each school category (52–79%); however, 59% (335/570) were committed to a single sports item in a certain school period, even if they changed sports items in the next school category (Figure S1). For example, a participant committed to basketball in upper elementary school and volleyball in junior high school, that is, the participant committed to a single sports item in a certain school period. The highest proportion of those with multiple sports experiences was found in upper elementary school (30%) among school categories, accounting for 24%, 5%, and 1% of two, three, and four sports items, respectively. On the other hand, in junior high and high schools, 90% and 99% of the participants played only one sport. The weekly hours spent in sports significantly increased as the students went to the higher school category (Table 2, Figure 2).

The mean (SD) sports-starting age in male participants (7.9 (2.6) years) was younger than in females (8.7 (3.3) years, *p* < 0.01). The sports duration for male participants (median: 6.5 years, IQR: 3.7 years) was significantly longer than that for female participants (median: 4.5 years, IQR: 4.0 years, *p* < 0.01). Single-sport duration ranged between 0.2 and 12.0 years (median: 6.0 years, IQR: 5.0 years). The proportion of those experienced in city-level competitions (56%) was significantly more than those experienced in prefectural- (38%) or national-level (6%) competitions (*p* < 0.01). The number of sports items engaged in by all participants was 31 (Table S2).

### 3.3. Sports-Related Injuries

**Figure 3:**
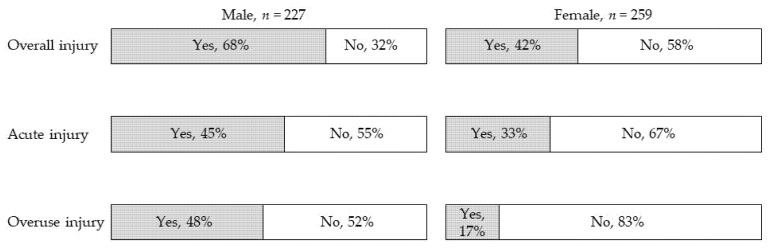
Prevalence of sports-related injuries in the lifetime. All proportions with injuries in males were significantly higher than those in females (*p* < 0.01).

**Figure 4:**
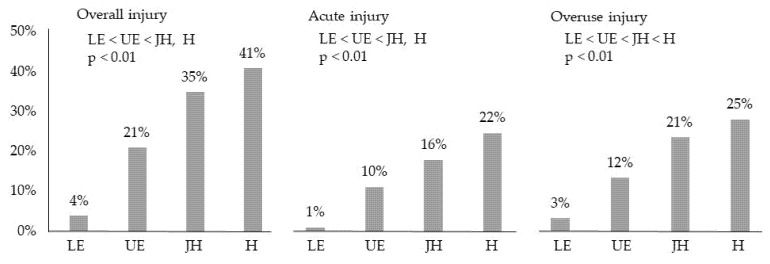
Prevalence of sport-related injuries in each school category. LE: Lower Elementary School, UE: Upper Elementary School, JH: Junior High School, H: High School.

Of the 486 participants with experience in sports activities in their lifetime, 263 (54%) experienced acute or overuse injuries. Male participants (*n* = 155, 68%) became injured significantly more than female participants (*n* = 108, 42%) (Figure 3). The proportions of acute or overuse injuries in male participants were also higher than those in female participants. The proportions of injured participants significantly differed by school categories: lower elementary school (4%) < upper elementary school (21%) < junior high school (35%) and high school (41%) (Figure 4).

The injuries particularly occurred on lower and upper extremities, and the proportions were significantly higher than those in the other body parts: lower extremities (*n* = 164, 63% of those with any injuries), upper extremities (*n* = 130, 50%) > lower back (*n* = 70, 27%) > head (*n* = 11, 4%), chest (*n* = 8, 3%), upper back (*n* = 3, 1%), and hip (*n* = 3, 1%) (*p* < 0.01).

The duration to complete recovery ranged from 0.1 to 120 months (median: 3 months, IQR: 17 months). The number of those who returned to sports before complete recovery was 141 (54% of those with any injuries). One hundred participants (38%) had re-injuries.

## 4. Discussion

This descriptive study aimed to investigate sports specialization and sports-related injuries. We found that the proportion of those participating in multiple sports was the highest in upper elementary school (30%). However, only 10% in junior high and 1% in high school participated in multiple sports.

A Finnish cohort study [14] reported that 61% of high school students participated in two or more sports activities. The proportion in Japan seems to be enormously smaller than that in Finland. In the United States, early specialization is popular because many high school athletes, their parents, and coaches hope to secure a college sports/athletic scholarship or even a contract with a professional sports team after school graduation; however, only 0.02%–0.46% achieve the latter goal [10]. Our results are comparable to those of a previous study [10]. Such early specialization is designed to make elite athletes before puberty [9]. Some athletes participating in specific sports (e.g., gymnastics, figure skating, and swimming) choose early specialization because their performance peaks at younger ages than those participating in other sports [6,9,10]. However, they are not the majority. In most sports, late specialization with early diversification would lead to success [9,15]. Young athletes (14.2 (1.6) years) believe that sports specialization makes them better in the sport and join high school or college teams [8]. Although we were unable to confirm whether the participants in this study believed the same things or not, we found unidentified sports diversifications in junior high and high schools.

A longitudinal clinical case–control study demonstrated that female athletes who tend to adopt individual and technical sports involving repetitive movement patterns (e.g., serving in volleyball) were more likely to become injured than male athletes [4]. The findings of this previous research contradict our findings, which demonstrated that male participants became injured more than the female participants (68% vs. 42% in overall injury, for example). No significant differences in sports item adoption were found between males and females (Table S2). Therefore, further research is needed to elucidate the reason for this contradiction.

In this study, the proportion of injuries during the high school period was 41%. Although it is difficult to compare directly, this proportion was comparable to that reported in a US study of 75,298 high school students among 10 sports items, in which the proportion of injury ranged from 13.2% (baseball in a male) to 50.0% (football in a male) [21]. More than half of our participants with any injuries (54%) returned to sports before complete recovery. Further, 38% had re-injuries. An earlier study demonstrated that athletes with a previous injury were likely to become injured again during a follow-up period of 36 months [4]; therefore, researchers should propose a preventive program for re-injury. Returning to sports before complete recovery should be avoided in the preventive program. Education about re-injury to athletes, their parents, and coaches can be significant. These results underscore the need for effective prevention and recovery strategies, even among non-elite athletes.

There are several significant limitations in this study. First, our survey creates a recall bias, which may produce potentially biased data due to under- or over-reporting sports experiences and sports-related injuries. Second, we did not collect data on modifiable variables, such as fitness level, precise training volume, and various psychological factors [2], explaining the injury. Third, we did not take into account the types and severity of injuries. For example, in our study, the number of head injuries was 11 (4%); this was significantly lower than that of upper or lower extremity injuries. However, a biomechanics study demonstrated that even minor injuries of the head might hurt the brain [22], which also requires a preventive program prior to injuries on other body parts.

Despite the abovementioned limitations, there are considerable strengths. First, we included non-medical treatments to achieve a broad view of sports-related injuries. Another strength was that we divided injuries into acute and overuse ones as the respective injury mechanisms are different. Therefore, this study has practical implications.

The participants in this study were non-elite athletes; therefore, our findings may not extrapolate to elite athletes. However, as the number of non-elite athletes is much larger than that of their elite counterparts, the study findings can benefit non-elite athletes. They provide actionable insights for sports- and school-related policymakers.

## 5. Conclusions

This study clarified a slight variation in sports items, particularly in junior high and high schools, which demonstrates 13 years as the age of beginning specialization in a single sport. More than half of non-elite athletes experienced sports-related injuries. The injuries were observed frequently in males and those in junior high and high schools. Future studies should investigate the association between sports specialization and sport-related injuries.

## Figures and Tables

**Table 1:** Participants’ characteristics.

Characteristic	Male (*n* = 237)	*p*-Value	Female (*n* = 333)	*p*-Value
Age (years)	20.3 ± 1.4	―	20.0 ± 1.2	―
Body height (m)	1.72 ± 0.06 *	<0.01	1.58 ± 0.05	0.80
Body weight (kg)	65.1 ± 9.0 *	<0.01	49.4 ± 5.5 ^†^	0.01
Body mass index (kg/m^2^)	22.0 ± 2.9 *	<0.01	19.8 ± 1.9 ^†^	<0.01

* Significantly larger than the national average [20]. ^†^ Significantly smaller than the national average [20].

**Table 2:** Characteristics of sports items and weekly hours in sports.

School Category	Number of Sports Items	Overall	Male	Female	Average Weekly Hours in Sports
Lower Elementary School	Total	296 [52%]	153 [65%] *^a^	143 [43%] *^a^	6.1 ± 5.5 *^c^
	One	228 (77%) *^b^	117 (77%) *^b^	111 (78%) *^b^	5.8 ± 5.4
	Multiple	68 (23%)	36 (23%)	32 (22%)	6.8 ± 5.7
Upper Elementary School	Total	354 [62%] *^a^	190 [80%] *^a^	164 [49%]	8.4 ± 6.1 *^c^
	One	248 (70%) *^b^	136 (72%) *^b^	112 (68%) *^b^	8.3 ± 6.2
	Multiple	106 (30%)	54 (28%)	52 (32%)	8.6 ± 5.7
Junior High School	Total	449 [79%] *^a^	222 [94%] *^a^	227 [68%] *^a^	15.9 ± 6.4 *^c^
	One	404 (90%) *^b^	193 (87%) *^b^	211 (93%) *^b^	16.0 ± 6.4
	Multiple	45 (10%)	29 (13%)	16 (7%)	15.9 ± 6.9
High School	Total	324 [57%] *^a^	196 [83%] *^a^	128 [38%] *^a^	16.4 ± 7.4 *^c^
	One	321 (99%) *^b^	193 (98%) *^b^	128 (100%) *^b^	16.4 ± 7.4
	Multiple	3 (1%)	3 (2%)	0 (0%)	10.7 ± 6.4
Lifetime *^d^	Total	486 [85%] *^a^	227 [96%] *^a^	259 [78%] *^a^	11.8 ± 5.4
	One	142 (29%) *^b^	52 (23%) *^b^	90 (35%) *^b^	12.5 ± 5.5 *^b^
	Multiple	344 (71%)	175 (77%)	169 (65%)	9.6 ± 4.7

Percentages are proportions to the participants in each school category and each sex category. *^a^: Significantly different between those with and without sports experience (*p* < 0.05). *^b^: Significantly different between one and multiple sports items (*p* < 0.05). *^c^: Junior High School and High School > Lower Elementary School and Upper Elementary School (*p* < 0.05). *^d^: Through Lower Elementary School to High School.

## Data Availability

The data presented in this study are available on request from the corresponding author. The data are not publicly available in compliance with the investigation confidential.

## Supplementary Materials

The following are available online at https://www.mdpi.com/article/10.3390/ijerph18147369/s1, Table S1: Questions about sports experiences and sports-related injuries, Table S2: Number (%) of those with experience and those with injury in each sports item, and Figure S1: Number of sports items adopted by each participant in each school category.
