# Supplementary material for: Sports Specialization and Sports-Related Injuries in Japanese School-Aged Children and Adolescents: A Retrospective Descriptive Study
Source: Int J Environ Res Public Health. 2021 Jul 9;18(14):7369. doi: 10.3390/ijerph18147369 (PMC8307530; doi:10.3390/ijerph18147369)
Supplement: Supplementary file 1 [file ijerph-18-07369-s001.zip › Supplementary Material (Figure S1).pdf]

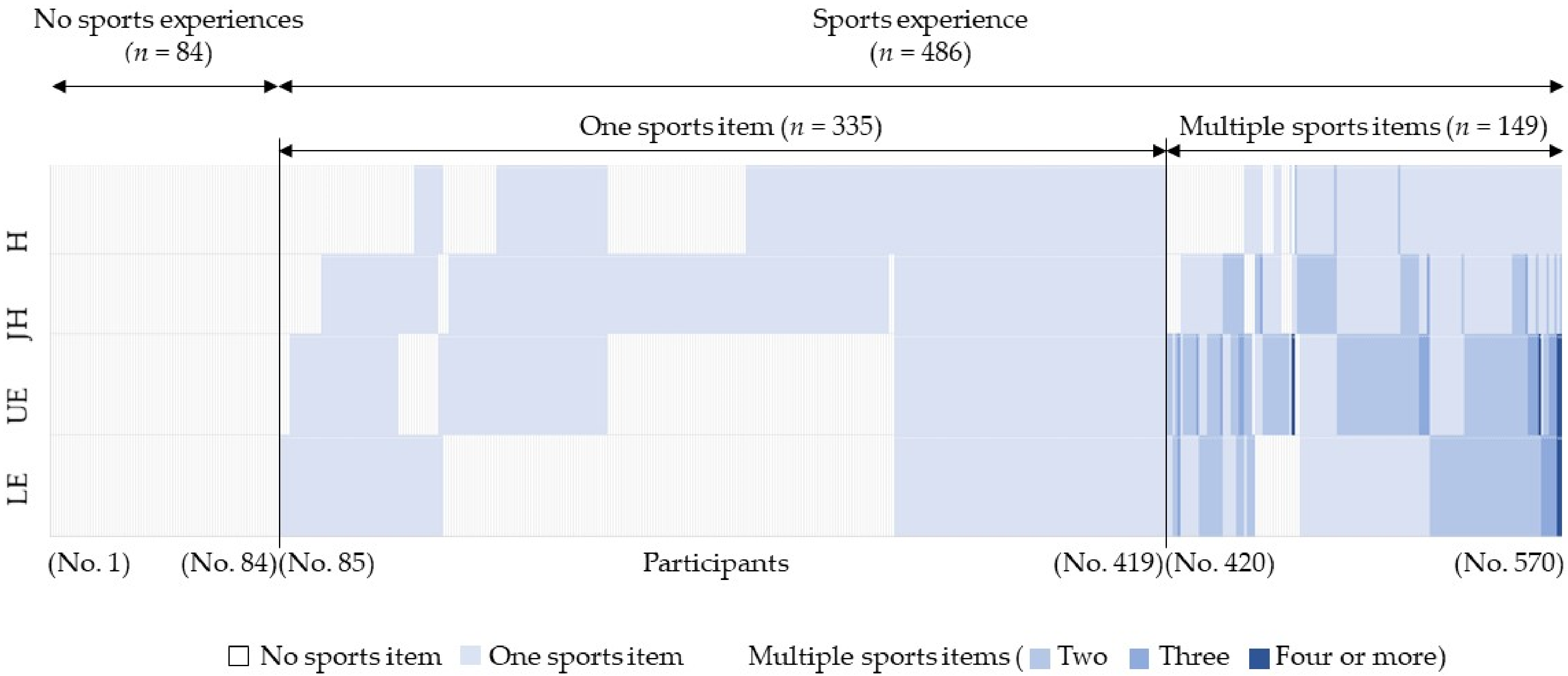

LH: Lower Elementary School; UE: Upper Elementary School; JH: Junior High School; H: High School. The horizontal line means the study participants (n = 570) arranged to show 59% (n = 335) adopted in only one sports item in a certain school category (from No. 85 to 419).

Figure S1. Number of sports items adopted by each participant in each school category.
