# Supplementary material for: Sports Specialization and Sports-Related Injuries in Japanese School-Aged Children and Adolescents: A Retrospective Descriptive Study
Source: Int J Environ Res Public Health. 2021 Jul 9;18(14):7369. doi: 10.3390/ijerph18147369 (PMC8307530; doi:10.3390/ijerph18147369)
Supplement: Supplementary file 1 [file ijerph-18-07369-s001.zip › Supplementary Material (Table S1).pdf]

Table S1. Questions about sports experiences and sports-related injuries

1. Have you ever participated in any organized sports activities such as extra-curricular activities (Bukatsu) or sports club activities? If yes, please proceed to the next question. If no, please stop responding to the questionnaire and hand it to the research staff. <Yes/No>

2. Please fill in the table about your sports experiences from elementary school to high school. You may refer to the example below. The term "sports experiences" is defined as any activity in sports organizations such as "Bukatsu" and sports clubs. However, the term excludes physical education classes in schools and recreational activities. The term "sports-related injuries" is defined as acute injuries or overuse injuries, which occurred during (due to) sports activities so that you could not participate in any sports activities for one day or longer.

Example

**Sports Experience**	**Age**	**Sports-Related Injuries**
Swimming	9	
Swimming	10	
Basketball	11	Jumper's knee
Athletics	12	Jumper's knee, Back pain
Athletics	13	Jumper's knee, Back pain

3. Please describe in detail your sports experiences. Similar to the example below, please use one column for one experience.

(1) If you participated in seasonal sports such as skiing for multiple years, fill the entire month. For example, if you participated in an activity for two months for two years, the total number of months is 4.
(2) If you require more columns, please use the backside of this page and add columns.

Example

Sport item [baseball]
Position, specific item, etc. [pitcher, infielder]
[x] Bukatsu / [ ] Sports club
Duration [6] years [0] months
Frequency [2] times/week or<br>[ ] times/month
Time [7] hours/week or<br>[ ] hours/month
Best tournament<br>[ ] National / [x] Prefectural / [ ] City

4. Please describe in detail your sports-related injuries. Similar to the example below, please use one column for one injury.

(1) If you do not remember the proper diagnosis, please describe the injury in as much detail as possible.
(2) In the bracket for the duration, please fill the number of months required for complete recovery.
(3) If an injury has not been treated yet, please count the length of duration from the month of injury to the month of high school graduation.

Example

Injury name [Fracture, the middle finger of the right hand]
[x] Acute / [ ] Overuse
Has a physician diagnosed you? [x] Yes / [ ] No
Completed cured? [x] Yes / [ ] No
Duration [1] years [2] months
If returned to the sport item, [ ] before / [x] after complete recovery.
Re-injured? [ ] Yes / [x] No

5. Please fill in your characteristics below.

(1) Sex:
(2) Age (years):
(3) Height (cm):
(4) Weight (kg):
(5) Sports-starting age (years):
