# Supplementary material for: Sports Specialization and Sports-Related Injuries in Japanese School-Aged Children and Adolescents: A Retrospective Descriptive Study
Source: Int J Environ Res Public Health. 2021 Jul 9;18(14):7369. doi: 10.3390/ijerph18147369 (PMC8307530; doi:10.3390/ijerph18147369)
Supplement: Supplementary file 1 [file ijerph-18-07369-s001.zip › Supplementary Material (Table S2) (2).pdf]

Table S2. Number (%) of those with experience and those with injury in each sports item.

	Sports item	Those with experience, n (%*a)						Those with injury, n (%*b)					
		Overall		Male		Female		Overall		Male		Female	
		(n = 486)		(n = 227)		(n = 259)		(n = 262, 54%)		(n = 154, 68%)		(n = 108, 42%*)	
1	Swimming *c	189	(39%)	81	(36%)	108	(42%)	15	(8%)	7	(9%)	8	(7%)
2	Rubber Baseball	94	(19%)	91	(40%)	3	(1%)	49	(52%)	48	(53%)	1	(33%)
3	Basketball	84	(17%)	31	(14%)	53	(20%)	42	(50%)	17	(55%)	25	(47%)
4	Track and Field	77	(16%)	40	(18%)	37	(14%)	38	(49%)	22	(55%)	16	(43%)
5	Soft Tennis	76	(16%)	25	(11%)	51	(20%)	14	(18%)	7	(28%)	7	(14%)
6	Tennis	68	(14%)	32	(14%)	36	(14%)	14	(21%)	11	(34%)	3	(8%*)
7	Volleyball	64	(13%)	18	(8%)	46	(18%)	33	(52%)	11	(61%)	22	(48%)
8	Football	62	(13%)	55	(24%)	7	(3%)	21	(34%)	20	(36%)	1	(14%)
9	Baseball	56	(12%)	55	(24%)	1	(0%)	36	(64%)	36	(65%)	0	(0%)
10	Softball	50	(10%)	23	(10%)	27	(10%)	18	(36%)	7	(30%)	11	(41%)
11	Table Tennis	44	(9%)	16	(7%)	28	(11%)	6	(14%)	2	(13%)	4	(14%)
12	Badminton	38	(8%)	8	(4%)	30	(12%)	8	(21%)	3	(38%)	5	(17%)
13	Dance Sport	35	(7%)	6	(3%)	29	(11%)	14	(40%)	2	(33%)	12	(41%)
14	Gymnastics	27	(6%)	14	(6%)	13	(5%)	6	(22%)	1	(7%)	5	(38%)
15	Karatedo	25	(5%)	15	(7%)	10	(4%)	8	(32%)	6	(40%)	2	(20%)
16	Handball	21	(4%)	12	(5%)	9	(3%)	10	(48%)	7	(58%)	3	(33%)
17	Kendo	20	(4%)	8	(4%)	12	(5%)	6	(30%)	3	(38%)	3	(25%)
18	Rhythmic Gymnastics	19	(4%)	1	(0%)	18	(7%)	1	(5%)	0	(0%)	1	(6%)
19	Kyudo	15	(3%)	4	(2%)	11	(4%)	3	(20%)	2	(50%)	1	(9%)
20	Judo	9	(2%)	8	(4%)	1	(0%)	4	(44%)	3	(38%)	1	(100%)
21	Ballet	8	(2%)	0	(0%)	8	(3%)	2	(25%)	0	—	2	(25%)
22	Dodgeball	7	(1%)	4	(2%)	3	(1%)	4	(57%)	3	(75%)	1	(33%)
23	Rugby Football	5	(1%)	5	(2%)	0	(0%)	4	(80%)	4	(80%)	0	—
24	Baton Twirling	5	(1%)	0	(0%)	5	(2%)	0	(0%)	0	—	0	(0%)
25	Rowing	3	(1%)	2	(1%)	1	(0%)	0	(0%)	0	(0%)	0	(0%)
26	American Football	2	(0%)	2	(1%)	0	(0%)	2	(100%)	2	(100%)	0	—
	Others (5 items) *d	5	(1%)	0	(0%)	5	(2%)	2	(40%)	0	—	2	(40%)

*a: Percentages are proportions of those with experience to the overall, male, and female participants. *b: Percentages are proportions of those with injury to those with experience. *c: The most popular pair of sports experienced by the participants was swimming and basketball (n = 40), followed by a pair of swimming and tennis (n = 33) and pairs of swimming and track and field/rubber-ball baseball (n = 32, respectively). *d: Cycling (n = 2), Aikido (n = 1), Fencing (n = 1), Figure Skating (n = 1), Wrestling (n = 1). *: Significantly different from male participants.
